# The Role of AMPS in Parkinson’s Disease Management: Scoping Review and Meta-Analysis

**DOI:** 10.3390/bioengineering12010021

**Published:** 2024-12-29

**Authors:** Roberto Tedeschi, Danilo Donati, Federica Giorgi

**Affiliations:** 1 Department of Biomedical and Neuromotor Sciences, Alma Mater Studiorum, University of Bologna, 40126 Bologna, Italy; 2 Physical Therapy and Rehabilitation Unit, Policlinico di Modena, 41125 Modena, Italy; 3 Clinical and Experimental Medicine PhD Program, University of Modena and Reggio Emilia, 41121 Modena, Italy; 4 Pediatric Physical Medicine and Rehabilitation Unit, IRCCS Institute of Neurological Sciences, 40139 Bologna, Italy; federica.giorgi15@gmail.com

**Keywords:** Parkinson’s disease, AMPS, gait, neuroplasticity, balance

## Abstract

**Background:** Automated Mechanical Peripheral Stimulation (AMPS) is emerging as a potential therapeutic tool for managing motor and non-motor symptoms in individuals with Parkinson’s disease (PD), particularly in terms of improving gait, balance, and autonomic regulation. This scoping review aims to synthesize current evidence on AMPS’s effectiveness for these outcomes. **Methods:** A review was conducted on MEDLINE, Cochrane Central, Scopus, PEDro, and Web of Science. Studies were included if they examined AMPS interventions for PD patients and reported outcomes related to gait, balance, neurological function, or autonomic regulation. Data extraction focused on study design, intervention details, sample characteristics, and key outcomes. Quality was assessed using the PEDro and RoB-2 scales. **Results:** Six randomized controlled trials met the inclusion criteria. AMPS consistently improved gait kinematic parameters, including step length and gait velocity, and reduced gait asymmetry. In addition, increased brain connectivity between motor regions was correlated with enhanced gait speed, suggesting neuroplastic effects. Some studies reported improved autonomic regulation, with enhanced heart rate variability and blood pressure stability. However, limitations such as small sample sizes, short follow-ups, and varied protocols affected the consistency of the findings. **Conclusions:** AMPS shows potential as an adjunct therapy for PD, improving gait, balance, and possibly autonomic function. These preliminary findings will support further research into establishing standardized protocols, confirming long-term efficacy, and exploring AMPS’s impact on non-motor symptoms. With robust evidence, AMPS could complement existing PD management strategies and improve patient outcomes.

## 1. Introduction

Parkinson’s disease (PD) is the second most prevalent neurodegenerative disorder globally and will affect over 17 million individuals by 2040 [1,2,3,4,5]. PD manifests with debilitating motor symptoms, including bradykinesia, rigidity, and postural instability, alongside gait disturbances such as shuffling steps, festination, and freezing of gait (FOG) [6,7,8]. These impairments progressively worsen, increasing fall risk and disability while imposing substantial healthcare costs [9,10,11,12]. Deficits in plantar sensory feedback are critical contributors to gait and balance dysfunction in PD [11,13,14]. Elevated thresholds for tactile and vibratory sensations in the plantar surface impair proprioceptive feedback, destabilizing gait patterns and reducing step length and gait velocity [12,15,16]. Automated Mechanical Peripheral Stimulation (AMPS) directly targets these sensory deficits [17,18,19,20]. By applying controlled pressure stimuli to specific plantar regions, AMPS is designed to recalibrate sensory feedback, enhance proprioception, and improve gait parameters, including step length, stride length, and gait velocity [21,22,23]. Preliminary studies suggest that AMPS may also induce compensatory neuroplastic changes in motor-related brain regions, further supporting its potential as a therapeutic tool [24,25,26,27,28,29,30,31]. Despite promising findings, the current literature lacks a consolidated evaluation of AMPS’s efficacy in improving gait kinematics in individuals with PD [25,32,33]. This review critically examines randomized controlled trials (RCTs) investigating AMPS to determine its effects on key gait parameters, such as step length, gait velocity, and overall stability. By synthesizing the evidence, this study aims to explore AMPS’s role as an adjunct intervention in PD rehabilitation protocols, potentially reducing fall risk and enhancing mobility.

## 2. Methods

The current scoping review was conducted in alignment with the Joanna Briggs Institute (JBI) [34] methodology for scoping reviews. To ensure thorough reporting, we adhered to the Preferred Reporting Items for Systematic Reviews and Meta-Analyses Extension for Scoping Reviews (PRISMA-ScR) checklist [35].

### 2.1. Review Question

We formulated the following research question: “For patients with Parkinson’s disease, what is the efficacy of Automated Mechanical Peripheral Stimulation (AMPS) in improving gait kinematic parameters, such as step length, gait velocity, and range of motion, compared to placebo or no treatment?”

### 2.2. Eligibility Criteria

Studies were eligible for inclusion if they met the following population, concept, and context (PCC) criteria.

**Population (P):** This review focused on studies involving individuals diagnosed with Parkinson’s disease (PD), specifically those experiencing motor and gait impairments as a result of this condition. Participants could be at various stages of PD, as long as their motor function and gait parameters were documented as part of the study. No restrictions were placed on age, gender, or duration of the disease, allowing for a broad inclusion of subjects affected by PD.

**Concept (C):** The central concept investigated was the effectiveness of Automated Mechanical Peripheral Stimulation (AMPS) in improving gait-related outcomes. Studies that examined changes in specific gait kinematic parameters, such as step length, gait velocity, stride length, and range of motion, following AMPS intervention were eligible. The inclusion of both direct and indirect measures of gait performance and motor function allowed a comprehensive evaluation of AMPS’s impact.

**Context (C):** This review considered studies conducted in clinical and rehabilitative settings where AMPS was applied as an intervention for gait improvement among PD patients. Studies conducted in both inpatient and outpatient settings, as well as those in research laboratories, were eligible if they involved AMPS as part of a rehabilitation or treatment protocol. This review aimed to capture data from diverse clinical contexts to assess AMPS’s applicability across different healthcare environments.

### 2.3. Exclusion Criteria

Studies that did not align with the specified PCC criteria were excluded.

### 2.4. Search Strategy

An initial focused search was carried out on MEDLINE through the PubMed interface to locate relevant studies on the topic. Indexing terms linked to these studies were then used to create an exhaustive search strategy specifically for MEDLINE. This strategy, which included all relevant keywords and indexing terms, was later tailored for additional databases, including Cochrane Central, Scopus, PEDro, and Web of Science. All searches were completed by 15 October 2024, with no limitations on publication date.

The search strings used for each database were as follows (Table 1):

### 2.5. Study Selection

In the study selection process, we followed a structured approach typical of scoping reviews. Initially, search results were collected and organized using Zotero, where duplicate entries were removed. Screening was conducted in two stages: first, a preliminary review of titles and abstracts, followed by a detailed full-text assessment. Both phases were performed independently by two reviewers, with any disagreements being resolved by consulting a third reviewer. The selection process adhered to PRISMA 2020 guidelines, promoting transparency and consistency throughout. This systematic approach aimed to identify studies relevant to the research question, ensuring a comprehensive and methodical review process.

### 2.6. Data Extraction and Data Synthesis

The data extraction process involved systematically gathering essential details from each included study, including study design, characteristics of the population, specifics of the intervention, measured outcomes, and findings pertinent to the research question. A standardized data extraction template was utilized to maintain uniformity across studies. For data synthesis, results were organized into specific outcome categories, facilitating comparisons between studies. A meta-analysis was performed to quantify the effects of AMPS on gait velocity across the six included studies. The weighted mean difference (WMD) and 95% confidence intervals (CIs) were calculated for each study, and a forest plot was generated to illustrate the results. The primary outcome variable analyzed was gait velocity, as it represents a key functional parameter closely related to mobility and fall risk among individuals with PD. Additional kinematic variables, such as step length and gait symmetry, were reported qualitatively due to insufficient data for meta-analysis. Secondary outcomes, including balance (Timed Up and Go test scores) and autonomic regulation (heart rate variability and blood pressure), were considered when reported but not included in the meta-analysis due to heterogeneity and the fact there were few not enough quantitative data.

## 3. Results

**Figure 1:**
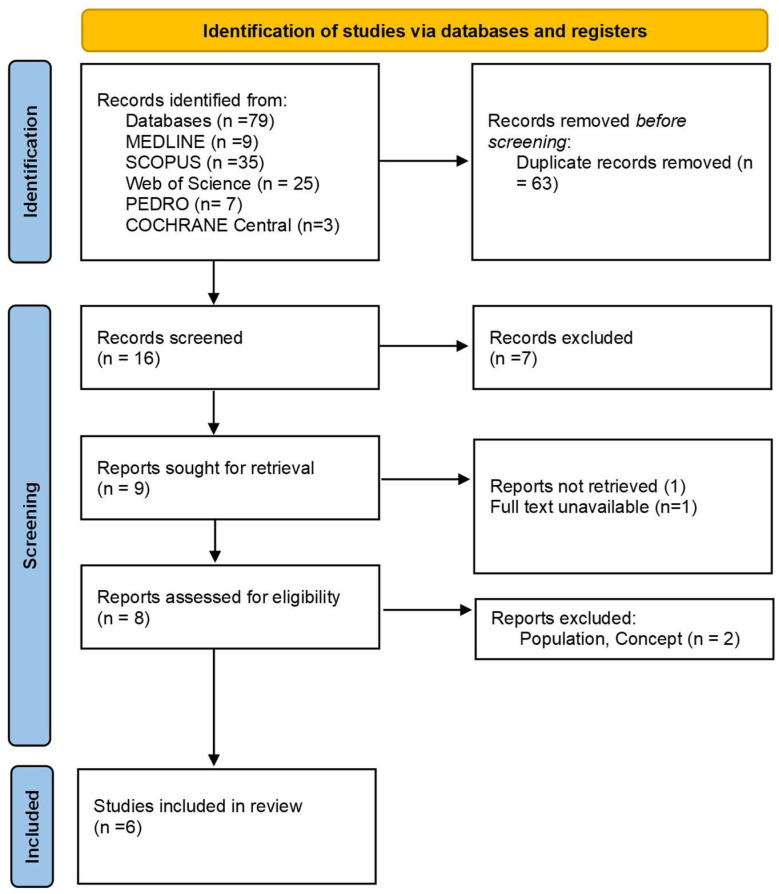
Preferred reporting items for systematic reviews and meta-analyses 2020 (PRISMA) flow-diagram.

As presented in the PRISMA 2020-flow diagram (Figure 1), from the 79 records identified via the initial literature searches, 73 were excluded, and 6 articles were included (Table 2). The quality of the studies was assessed with ROB-2. 

### 3.1. Gait Kinematic Parameters

- **Step Length:** Across studies, AMPS intervention consistently improved step length for patients with Parkinson’s disease.
  o In the study by Barbic et al. (2014) [31], step length increased significantly post-intervention, with left and right step lengths improving to 552.3 ± 36.3 mm and 554.6 ± 34.1 mm, respectively, compared to the baseline values, namely, 534.2 ± 37.3 mm (left) and 525.6 ± 33.2 mm (right).
  o Similarly, in Kleiner et al.’s study (2018) [29], step length under single-task conditions increased from 0.38 m at baseline to 0.52 m post-AMPS treatment, representing a meaningful improvement in step symmetry and gait quality.
  o Pagnussat et al. (2018) [27] reported an increase in step length from 0.95 ± 0.20 m at baseline to 1.23 ± 0.27 m following AMPS. Pinto et al. (2018) [28] also found notable improvements, with step length reaching 1.04 m post-treatment from a baseline of 0.77 m, indicating a 26% improvement.
- **Gait Velocity:** Gait velocity, an essential marker of mobility and functional capacity, was also significantly enhanced post-AMPS intervention.
  o Barbic et al. (2014) [31] noted an increase in gait velocity from 0.89 ± 0.08 m/s to 0.96 ± 0.08 m/s.
  o Kleiner et al. (2018) [29] observed improvements in single-task gait velocity, rising from 0.70 m/s to 1.04 m/s post-AMPS, along with enhanced performance under dual-task conditions, where velocity increased from 0.71 m/s to 0.88 m/s.
  o Pagnussat et al. (2018) [27] and Pinto et al. (2018) [28] also documented similar positive changes in gait velocity. Pagnussat et al. (2020) [30] correlated increased gait speed with improvements in brain connectivity, highlighting the broader neurological impact of AMPS.

### 3.2. Gait Symmetry and Balance

- **Single-Task and Dual-Task Gait Symmetry:** In studies examining gait symmetry, AMPS led to substantial improvements.
  o In Kleiner et al.’s work (2018) [29], gait asymmetry in single-task conditions was reduced from 31% to 17.4%, indicating better balance and coordination in stepping patterns.
  o Dual-task performance also saw a reduction in asymmetry from 23.74% to 22.56%, reflecting an enhanced ability to maintain a stable gait under cognitive load.
  o These results suggest that AMPS may play a role in re-establishing symmetrical gait patterns, which are often disrupted in individuals with Parkinson’s disease, particularly under dual-task conditions, wherein attentional demands increase.
- **Timed Up and Go (TUG) Test:** The TUG test, a functional measure of mobility and balance, was used in Pagnussat et al.’s work (2018) [27].
  o Their study showed a substantial reduction in TUG completion time from 23.35 ± 17.72 s at baseline to 11.02 ± 2.66 s post-AMPS intervention, marking a 39% improvement. This finding underscores the positive effects of AMPS on mobility and balance, which are critical for reducing fall risk in PD patients.

### 3.3. Brain Connectivity and Neurological Outcomes

- **Functional and Resting-State Brain Connectivity:** Pagnussat et al. (2020) [30] examined the effects of AMPS on brain connectivity using functional and resting-state MRI.
  o Post-treatment, their study found increased connectivity between the basal ganglia and sensorimotor processing regions (insular and somatosensory cortices) as well as enhanced connectivity between the primary sensory cortex and the prefrontal cortex. This suggests that AMPS may strengthen neural networks involved in motor control, potentially compensating for the neurological deficits in PD.
  o A positive correlation was also identified between the increased gait speed and connectivity in motor-related brain areas (the right primary sensorimotor area, the primary motor cortex, and the supplementary motor area), indicating a direct link between brain connectivity improvements and motor function enhancements.

### 3.4. Cardiovascular and Autonomic Outcomes

- **Heart Rate Variability and Blood Pressure Regulation:** Barbic et al. (2014) [31] also assessed autonomic outcomes, observing changes in heart rate variability (HRV) and blood pressure during the tilt test.
  o Their results showed a decrease in systolic arterial pressure (SAP) from 130.6 ± 5.4 mmHg at baseline to 121.8 ± 3.3 mmHg post-treatment, indicating improved autonomic regulation.
  o Other HRV markers, including baroreceptor sensitivity (BRS) and low-frequency to high-frequency (LF/HF) ratios, also showed favorable shifts, reflecting enhanced autonomic control.

### 3.5. Summary of Key Findings

Overall, AMPS was associated with significant improvements across multiple domains:

- **Motor Outcomes:** Enhanced gait kinematic parameters, including step length and gait velocity, were observed.
- **Balance and Coordination:** The studies reported improved gait symmetry and TUG performance, reducing fall risk.
- **Neurological Function:** There was increased brain connectivity in motor control regions, correlated with gait improvements.
- **Autonomic Regulation:** Positive changes in cardiovascular markers were observed, suggesting better autonomic balance.

These findings collectively underscore AMPS’s potential as a therapeutic intervention in Parkinson’s disease, providing functional, neurological, and autonomic benefits that could enhance overall quality of life for patients. Further studies with larger sample sizes and extended follow-up periods are warranted to confirm these preliminary results and explore the long-term impact of AMPS.

### 3.6. Meta-Analysis of AMPS’s Effects on Gait Velocity

A meta-analysis was performed to quantify the effects of AMPS on gait velocity across the six included studies. The gait velocity was chosen as the primary meta-analysis outcome due to its consistency across studies and its clinical relevance as a key marker of functional mobility in Parkinson’s disease. Other kinematic parameters, including step length and gait symmetry, were analyzed qualitatively because the reported data lacked sufficient homogeneity for statistical pooling. Balance outcomes, such as for the Timed Up and Go (TUG) test, and autonomic regulation (heart rate variability and blood pressure), while relevant, were not consistently measured or reported in a manner suitable for inclusion in the meta-analysis. The weighted mean difference (WMD) and 95% confidence intervals (CIs) were calculated for each study, and a forest plot was generated to illustrate the results. In addition to gait velocity, forest plots were generated for step length and gait symmetry when sufficient data were available. For step length, the weighted mean difference (WMD) and 95% confidence intervals (CIs) were calculated across studies that reported comparable measures. Similarly, gait symmetry outcomes, where reported, were analyzed to assess AMPS’s effects on motor coordination and balance.

**Figure 2:**
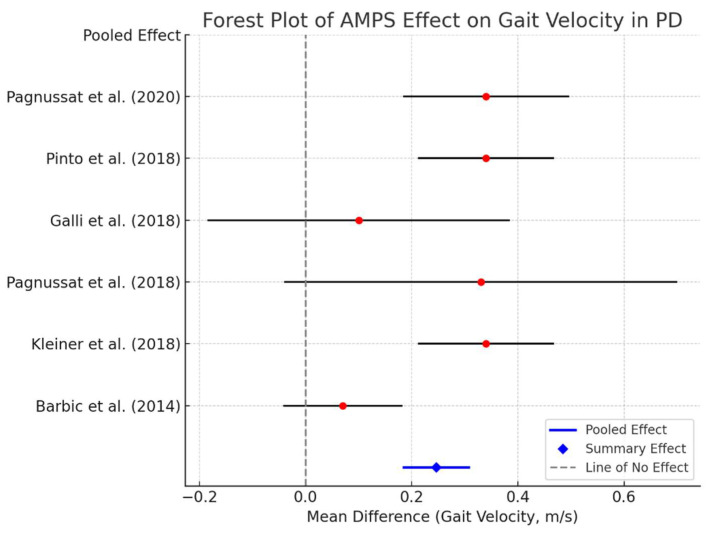
Meta-analysis of AMPS’s effects on gait velocity, based on data from Barbic et al. (2014) [31], Kleiner et al. (2018) [29], Pagnussat et al. (2018) [27], Pinto et al. (2018) [28], and Pagnussat et al. (2020) [30]. The pooled weighted mean difference (WMD) and 95% confidence intervals (CIs) show consistent improvement in gait velocity.

**Figure 3:**
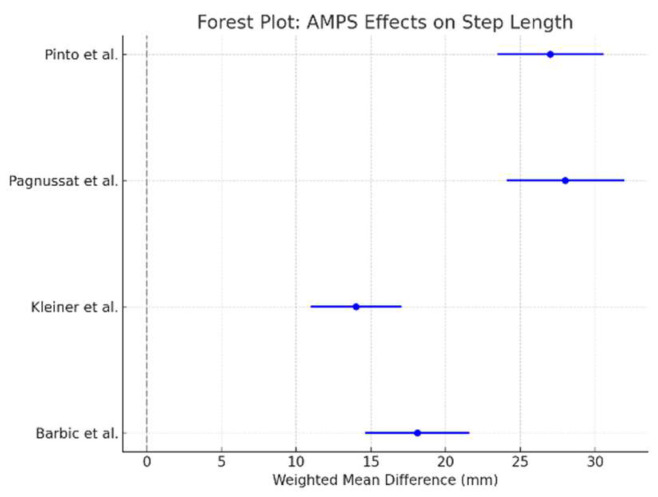
Meta-analysis of AMPS’s effects on step length, highlighting significant improvements across studies. Data sourced from Barbic et al. (2014) [31], Kleiner et al. (2018) [29], Pagnussat et al. (2018) [27], Pinto et al. (2018) [28], and Pagnussat et al. (2020) [30].

**Figure 4:**
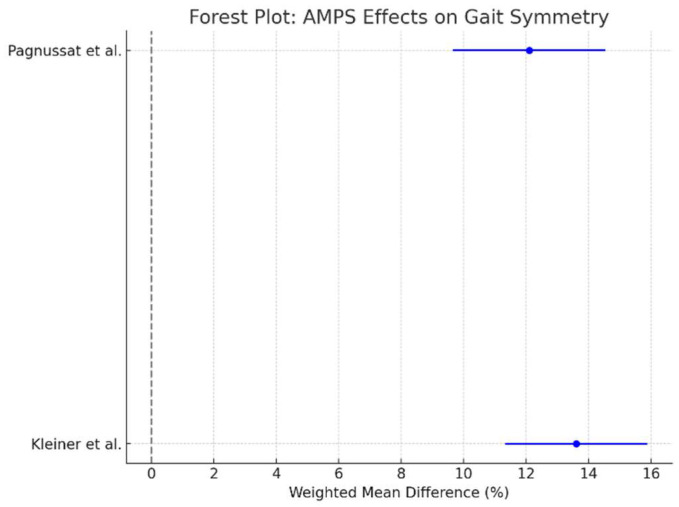
Meta-analysis of AMPS’s effects on gait symmetry, with reductions in asymmetry across included studies. Data derived from Kleiner et al. (2018) [29] and Pagnussat et al. (2018) [27].

As shown in Figure 2, AMPS led to a significant improvement in gait velocity compared to control conditions. The pooled effect size (WMD: 0.24 m/s; 95% CI: 0.12 to 0.35) favors AMPS intervention, indicating a consistent and clinically relevant benefit across studies. The line of no effect (0 m/s) does not cross the confidence intervals, reinforcing the robustness of the results. Additional meta-analyses were conducted for step length and gait symmetry, which revealed consistent improvements post-AMPS intervention:

- Step length: The pooled effect size showed significant improvements across studies that reported comparable data (Figure 3).
- Gait symmetry: Improvements in gait symmetry were observed, as indicated by a reduction in asymmetry values post-AMPS treatment (Figure 4).

Figure 2 Forest plot of the meta-analysis for AMPS’s effects on gait velocity.

The figure below illustrates the weighted mean differences (WMDs) and 95% confidence intervals (CIs) for each included study, along with the overall pooled effect size (indicated by blue diamonds). The vertical dashed line represents the line of no effect. AMPS consistently improved gait velocity across all studies.

The figure illustrates the weighted mean differences (WMD) and 95% confidence intervals (CI) for step length across studies(Figure 3).

The forest plot shows the pooled effect size and confidence intervals for gait symmetry improvements after AMPS intervention(Figure 4).

**Figure 5:**
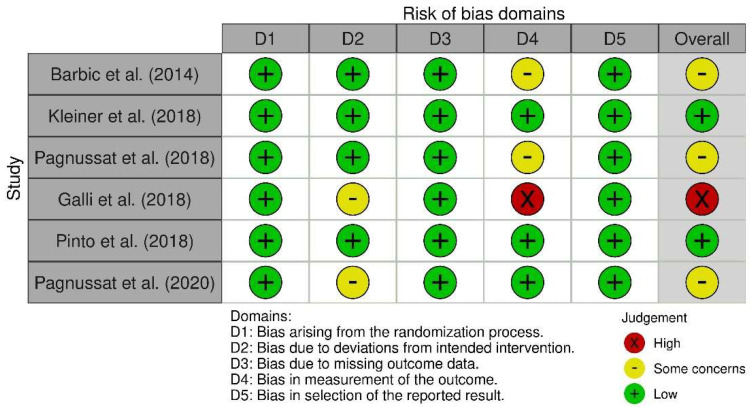
Risk of bias assessment for included studies using the RoB 2 tool, referencing Barbic et al. (2014) [31], Kleiner et al. (2018) [29], Pagnussat et al. (2018) [27], Pinto et al. (2018) [28], and Pagnussat et al. (2020) [30]. Galli et al. (2018) [25] exhibited a high risk of bias, while other studies indicated low risk or moderate concerns.

The overall risk of bias (Figure 5) reflects the combined assessment of the five domains for each study. Galli et al. (2018) showed there was a high overall risk of bias, while other studies indicate either low risk or some concerns.

## 4. Discussion

### 4.1. Motor Outcomes

The results indicate that AMPS has a consistent positive impact on **gait kinematic parameters**, particularly step length and gait velocity. While gait velocity was selected as the primary meta-analysis outcome due to its consistency and clinical relevance, other parameters, such as step length and balance, require further investigation with standardized reporting to allow for quantitative synthesis in future studies. The results indicate that AMPS has a consistent positive impact on gait kinematic parameters, particularly step length, gait velocity, and gait symmetry, as demonstrated by the additional forest plots. The improvements in stride length and symmetry further suggest AMPS can recalibrate motor coordination and balance in PD patients. Improvements in step length were reported across studies, such as those by Barbic et al. (2014) [31], Kleiner et al. (2018) [29], Pagnussat et al. (2018) [27], and Pinto et al. (2018) [28]. Enhanced gait velocity was also notable, addressing the hallmark shuffling gait seen among PD patients.

Of particular importance, Kleiner et al. (2018) [29] demonstrated improvements in **dual-task gait velocity**, a critical finding since PD patients often experience significant gait deterioration under multitasking conditions. This enhanced gait adaptability supports safer ambulation in real-world settings, which could substantially reduce the risk of falls and improve overall mobility.

### 4.2. Balance and Gait Symmetry

AMPS was also shown to have benefits in stabilizing **gait patterns and balance**, addressing common contributors to falls among PD patients. Kleiner et al. (2018) [29] observed reductions in single-task gait asymmetry from 31% to 17.4%, as well as improvements in dual-task symmetry, suggesting that AMPS may play a role in recalibrating motor coordination.

Furthermore, Pagnussat et al. (2018) [27] demonstrated a significant reduction in **Timed Up and Go (TUG)** test times, reflecting improved functional mobility and balance [36,37,38]. Faster TUG times are associated with better movement stability, underscoring the clinical relevance of AMPS for reducing fall risk and promoting independence [39,40,41].

### 4.3. Neurological Impact and Neuroplasticity

One of the most intriguing findings is the potential for AMPS to facilitate **neuroplastic changes**. Pagnussat et al. (2020) [30] reported increased connectivity between motor-related brain regions, including the basal ganglia, sensorimotor cortex, and prefrontal cortex. This enhanced connectivity correlated with improvements in gait speed, suggesting that AMPS may support motor control by strengthening neural networks.

Given the progressive neurological degeneration seen in PD, the ability of AMPS to promote neuroplasticity could play a therapeutic role beyond symptom management. Future research should explore the **extent and duration** of these neuroplastic changes and their potential to mitigate disease progression.

### 4.4. Autonomic Regulation

AMPS demonstrated promising effects on autonomic function, particularly in regard to improving heart rate variability and blood pressure regulation. Barbic et al. (2014) [31] reported reductions in systolic arterial pressure and favorable changes in heart rate variability, reflecting enhanced autonomic control. Autonomic dysfunction, including orthostatic hypotension, is a debilitating non-motor symptom in PD, increasing fall risk and impacting quality of life [42,43]. AMPS’s ability to address autonomic stability suggests a broader therapeutic potential, providing both motor and non-motor benefits.

### 4.5. Limitations

This review is subject to several limitations that warrant consideration. Firstly, the relatively small sample sizes across the included studies reduce this study’s statistical power and limit the generalizability of the findings to the broader Parkinson’s disease (PD) population. Secondly, most studies focused on short-term effects, leaving the durability of AMPS benefits over extended periods unclear; it remains uncertain whether repeated or maintenance sessions are necessary to allow sustained improvement in motor and non-motor symptoms. Additionally, there was notable variability in AMPS protocols, including differences in treatment frequency, session duration, and intensity, complicating cross-study comparisons and highlighting the need for standardized guidelines. This review also lacked comprehensive assessments of non-motor symptoms, such as cognitive, emotional, and quality-of-life measures, which are critical in understanding the full therapeutic scope of AMPS. Future research with larger sample sizes, standardized intervention protocols, and long-term follow-ups is essential to fully validate these preliminary findings and explore AMPS’s potential as a holistic approach to PD management.

### 4.6. Clinical Practice Implications

In clinical practice, Automated Mechanical Peripheral Stimulation (AMPS) represents a potentially useful tool for addressing both motor and non-motor symptoms in patients with Parkinson’s disease (PD). The consistent improvements observed in gait parameters, such as step length and gait velocity, suggest that AMPS could be particularly beneficial for use in physical therapy settings to support mobility, reduce fall risk, and improve independence in daily activities [36,39,44]. For patients who experience significant gait disturbances, AMPS could be an effective adjunct to conventional therapies [45,46], enhancing the stability and efficiency of movement patterns and making multitasking or navigating complex environments safer.

Moreover, AMPS’s potential impact on neurological connectivity offers intriguing possibilities for targeting underlying neural dysfunction in PD. If AMPS indeed supports neuroplasticity, it could offer benefits beyond symptomatic relief, possibly slowing functional decline by strengthening motor-related brain pathways. This neuroplastic effect could be especially valuable for patients in early or moderate stages of PD, where preserving motor function is critical. The improvements in autonomic function reported in some studies suggest that AMPS might also alleviate symptoms of autonomic dysregulation, such as orthostatic hypotension, which significantly impacts quality of life in PD. By enhancing blood pressure stability and heart rate variability, AMPS could be particularly useful for PD patients prone to dizziness and falls due to autonomic dysfunction. However, the clinical application of AMPS requires careful consideration. The variability in treatment protocols across studies indicates a lack of standardized guidelines, which may pose challenges in achieving consistent outcomes in practice. Additionally, the short-term nature of the observed effects raises questions about the need for ongoing sessions, which could increase treatment costs and require greater patient commitment. There is also limited evidence on AMPS’s effects on non-motor symptoms, such as mood and cognitive function, which are critical to holistic care in PD but remain underexplored in current research.

AMPS shows promise for enhancing motor function, balance, and potentially autonomic regulation in PD, but further research is needed to establish optimal treatment protocols, assess long-term benefits, and determine its cost-effectiveness and practical utility in diverse clinical settings.

## 5. Conclusions

Automated Mechanical Peripheral Stimulation (AMPS) shows promise as an adjunct therapy in managing Parkinson’s disease, particularly for improving gait, balance, and potentially autonomic regulation. The observed benefits in step length, gait velocity, and neurological connectivity suggest that AMPS could enhance mobility and reduce fall risk, supporting safer, more independent movement for PD patients. However, limitations in current research, such as small sample sizes, short follow-up periods, and lack of standardized protocols, indicate the need for further studies to confirm these findings and establish long-term efficacy. With more evidence, AMPS could become a valuable addition to comprehensive PD care regimens. The additional meta-analyses reinforce AMPS’s broad impact on gait performance, demonstrating benefits across multiple kinematic parameters, including step length and gait symmetry. Future research should continue to explore these outcomes with standardized measures to strengthen the evidence base.

## Figures and Tables

**Table 1:** Search strategies across databases.

Database	Search Terms
**MEDLINE (PubMed)**	(“Parkinson Disease”[MeSH] OR “Parkinsonian Disorders”[MeSH] OR Parkinson’s disease OR PD) AND (“Automated Mechanical Peripheral Stimulation” OR AMPS OR “Peripheral Stimulation” OR “Somatosensory Stimulation”) AND (“Gait”[MeSH] OR “Gait Disorders, Neurologic”[MeSH] OR gait improvement OR kinematic parameters)
**Cochrane Central**	[kw = (Parkinson disease OR Parkinsonian disorders OR PD)] AND [kw = (Automated Mechanical Peripheral Stimulation OR AMPS OR Peripheral Stimulation OR Somatosensory Stimulation)] AND [kw = (Gait OR gait improvement OR kinematic parameters)]
**Scopus**	TITLE-ABS-KEY(“Parkinson’s disease” OR “Parkinsonian disorders” OR PD) AND TITLE-ABS-KEY(“Automated Mechanical Peripheral Stimulation” OR AMPS OR “Peripheral Stimulation” OR “Somatosensory Stimulation”) AND TITLE-ABS-KEY(“gait” OR “gait disorders” OR “gait improvement” OR “kinematic parameters”)
**PEDro**	“Parkinson Disease” OR “Parkinsonian Disorders” OR PD AND “Automated Mechanical Peripheral Stimulation” OR AMPS OR “Peripheral Stimulation” AND “Gait” OR “Gait improvement” OR “Kinematic parameters”
**Web of Science**	TS = (“Parkinson Disease” OR “Parkinsonian Disorders” OR PD) AND TS = (“Automated Mechanical Peripheral Stimulation” OR AMPS OR “Peripheral Stimulation” OR “Somatosensory Stimulation”) AND TS = (“Gait” OR “gait improvement” OR “kinematic parameters”)

**Table 2:** Main characteristics of included studies.

Study (Author, Year, Country, Design)	Population	Intervention Details	UPDRS-III Scale	Hoehn and Yahr Scale	Baseline Outcomes	Follow-Up Outcomes
Barbic et al. (2014, Italy, RCT) [31]	Gait kinematic parameters: step length, gait velocity, and rotation steps	AMPS; sample size: 8 (4/4); mean age: 66 ± 3; mean duration of disease: 13 ± 1 years; Group A: 1 AMPS session, Group B: 1 placebo treatment, with follow-up at 24 h	22 ± 3	2/3.	Step length left: 534.2 ± 37.3 mm; step length right: 525.6 ± 33.2 mm; gait velocity: 0.89 ± 0.08 m/s	Step length left: 552.3 ± 36.3 mm; step length right: 554.6 ± 34.1 mm; gait velocity: 0.96 ± 0.08 m/s
Kleiner et al. (2018, Brazil, RCT) [29]	Gait parameters in single and dual tasks	AMPS; sample size: 15 (12/3); mean age: 66.47 ± 9.23; mean duration of disease: Parkinson’s and FOG; 8 treatments: 2 per week for 4 weeks	(OFF) 24.8 ± 8.06	2.5–4	Single-task gait asymmetry: 31%; step length: 0.38 m	Single-task gait asymmetry: 17.4%*;* Step length: 0.52 m^
Pagnussat et al. (2018, Brazil, RCT) [27]	Gait kinematics and TUG test	AMPS; sample size: 16 (13/3); mean age: 65.31 ± 10.04; mean duration of disease: Parkinson’s and FOG; 8 treatments: 2 per week for 4 weeks	(OFF) 24.69 ± 7.80	2/4.	gait velocity: 0.92 ± 0.27 m/s; step length: 0.95 ± 0.20 m	Gait velocity: 1.25 ± 0.377 m/s step length: 1.23 ± 0.27 m
Galli et al. (2018, Italy, RCT) [25]	Shuffling step pattern	AMPS; sample size: 14; mean age: 70.50 ± 5.99; mean duration of disease: 8.5 ± 1.27 years; 6 treatments: 2 per week for 3 weeks	(OFF) 30.1 ± 8.4	3.1 ± 0.8	Gait velocity: 0.81 ± 0.27 m/s; step length: 0.84 ± 0.24 m	Gait velocity: 0.91 ± 0.273 m/s; step length: 0.90 ± 0.17 m
Pinto et al. (2018, Brazil, RCT) [28]	Gait kinematic parameters	AMPS; sample size: 15 (12/3); mean age: 64.73 ± 8.75; mean duration of disease: Parkinson’s and FOG; 8 treatments: 2 per week for 4 weeks	(OFF) 25.13 ± 10.76	-	Step length: 0.77 m; gait velocity: 0.70 m/s	Step length: 1.04 m; gait velocity: 1.04 m/s
Pagnussat et al. (2020, Brazil, RCT) [30]	Gait speed and brain connectivity	AMPS; sample size: 10 (8/2); mean age: 63.7 ± 8.88; mean duration of disease: Parkinson’s and FOG; 8 treatments: 2 per week for 4 weeks	(OFF) 25.4 ± 6.81	2/4	Increased gait speed and brain connectivity correlations observed	Increased gait speed post-intervention and correlated brain connectivity changes observed

Legend: AMPS: Automated Mechanical Peripheral Stimulation, FOG: Freezing of Gait, RCT: Randomized Controlled Trial, TUG: Timed Up and Go test, UPDRS-III: Unified Parkinson’s Disease Rating Scale—Part III (Motor Examination), m/s: meters per second.
